# The Association between Diabetic Retinopathy and Levels of Ischemia-Modified Albumin, Total Thiol, Total Antioxidant Capacity, and Total Oxidative Stress in Serum and Aqueous Humor

**DOI:** 10.1155/2014/820853

**Published:** 2014-12-16

**Authors:** Kadir Kirboga, Ayse V. Ozec, Mustafa Kosker, Ayhan Dursun, Mustafa I. Toker, Huseyin Aydin, Haydar Erdogan, Aysen Topalkara, Mustafa K. Arici

**Affiliations:** ^1^Department of Ophthalmology, Cumhuriyet University School of Medicine, 58140 Sivas, Turkey; ^2^Department of Ophthalmology, Ulus State Hospital, Ankara, Turkey; ^3^Department of Biochemistry, Cumhuriyet University School of Medicine, 58140 Sivas, Turkey

## Abstract

*Purpose*. To investigate the oxidant and antioxidant status of patients with type 2 diabetes mellitus and nonproliferative diabetic retinopathy (DRP).* Methods*. Forty-four patients who had cataract surgery were enrolled in the study. We included 22 patients with DRP in one group and 22 patients in the control group. Samples of aqueous humor and serum were taken from all patients. Serum and aqueous ischemia-modified albumin (IMA), total thiol, total antioxidant capacity (TAC), and total oxidative stress (TOS) levels were compared in two groups.* Results*. Median serum IMA levels were 44.80 absorbance units in the DRP group and 40.15 absorbance units in the control group (*P* = 0.031). Median serum total thiol levels in the DRP group were significantly less than those in the control group (3051.13 and 3910.12, resp., *P* = 0.004). Mean TOS levels in the serum were 2.93 ± 0.19 in the DRP group and 2.61 ± 0.26 in the control group (*P* = 0.039). The differences in mean total thiol, TAC, and TOS levels in the aqueous humor and mean TAC levels in the serum were not statistically significant.* Conclusion*. IMA, total thiol, and TOS levels in the serum might be useful markers in monitoring the risk of DRP development.

## 1. Introduction

Diabetes mellitus (DM) is a common disease around the world. Diabetic retinopathy (DRP) is one of the main reasons of blindness [1, 2]. Microvascular and macrovascular damage developing in consequence of chronic hyperglycemia has a negative effect on the retina. One of the pathologic changes in DRP is retinal ischemia and oxidative stress-related neovascularization [3–5].

Ischemia-modified albumin (IMA) is a form of oxidatively modified albumin. It has been postulated that IMA is a new marker for ischemia [6, 7] and oxidative stress [8]. High serum IMA levels have been determined in patients with type 2 DM [9–12]. No research assessing IMA levels in the aqueous of patients with DRP has been found in the literature.

Thiol groups are one of the members of the antioxidant system as they have been revealed to devastate the reactive oxygen species (ROS) and other free radicals by enzymic and nonenzymic mechanisms [13, 14]. It has been recently advocated that genetic factors may also have an effect on the ROS system activity and ROS production [15]. It has been found that the exposure of proteins to oxidative stress resulted in decrease and functional defects in the thiol groups [16, 17]. Low serum total thiol levels have been detected in patients with type 2 DM [18]. But there was no study found in the literature assessing the total thiol levels in the serum and aqueous humor of patients with DRP.

Observing the tissue ischemia in patients with DM is vital in regulating perfusion impairment. Although the main risk factors (glycosylated hemoglobin concentration, duration of diabetes, and blood pressure) are thought to correlate with the incidence and progression of DRP, they can explain only a limited amount of the risk of developing these complications [19, 20]. But these factors are not efficacious in observing the amount of tissue ischemia caused by DM.

In this report, we examine the oxidant and antioxidant status of patients with type 2 DM who had nonproliferative DRP and compare them with those of age and sex matched patients in the control group.

## 2. Patients and Methods

Forty-four consecutive patients who underwent cataract surgery were enrolled in this study at the Cumhuriyet University School of Medicine from January 2012 to July 2012. At the beginning of the study, we conducted a preliminary study on 10 patients with type 2 DM in one group (DRP group) and 10 patients in the control group. Based on the obtained IMA and total thiol levels in the serum, the influence quantity was *d* = 0.87, and it was calculated that we need to include in the study at least 22 patients with type 2 DM and 22 patients in the control group to achieve 80% power at the level of *α* = 0.05. Patients were classified into two groups: 22 patients with type 2 DM and nonproliferative DRP were included in DRP group and 22 patients were included in the control group. This study was approved by the Ethical Committee of Cumhuriyet University School of Medicine (Sivas, Turkey) and it conforms to the provisions of the Declaration of Helsinki. Informed consent was obtained from all the patients participating in the study.

### 2.1. Exclusion Criteria for the Study

Patients with a history of ischemic artery disease (e.g., cerebrovascular disease, cardiovascular disease, deep vein thrombosis, or arterial occlusion) were excluded. Furthermore, patients with hepatic, renal, or cardiac inefficiency or electrocardiogram defects or with abnormal serum levels of albumin (<3.5 and >5.5 mg/dL), which may affect chemical analyses, were excluded.

Biomicroscopic eye examination was performed, and normal and red-free fundus images were consequently taken. Early Treatment Diabetic Retinopathy Study [21] criteria were applied in the diagnosis of DRP from the taken fundus images.

### 2.2. Collecting and Preserving of the Samples

#### 2.2.1. Aqueous Samples

Paracenteses were performed using a 27-gauge needle attached to an insulin syringe. The 100–150 *μ*L aqueous was aspirated.

#### 2.2.2. Blood Samples

Blood samples were collected from patients with type 2 DM and patients in the control group, following an overnight fast just prior to cataract surgery. Serum was separated from the cells by centrifugation at 2500 rpm for 15 minutes. Serum and aqueous samples were stored at −80°C until the analyses were performed for IMA, total thiol, TAC, and TOS.

### 2.3. Laboratory Analysis

#### 2.3.1. Ischemia-Modified Albumin Assay

IMA was analyzed by using an IMA ELISA kit with an autoanalyzer (ChemWell, Palm City, USA).

#### 2.3.2. Total Thiol Assay

Total thiol was analyzed using a total thiol assay kit from Rel Assay Diagnostics (Gaziantep, Turkey) with an autoanalyzer (Advia 2400 Chemistry System, Erlangen, Germany). A spectrophotometric analysis based on 2,2-dithiobisnitrobenzoic acid (DTNB) was used for thiol analysis. An aliquot of serum was mixed with Tris-EDTA buffer, and later DTNB was added. After a 15-minute incubation at room temperature, the absorbance was measured at 412 nm. A reagent blank without sample and a sample blank with methanol instead of DTNB were prepared in a similar manner. A GSH (50, 100, 250, and 500 *μ*mol/L) solution was used as calibrator. Thiol levels were expressed as mmol/L^−1^.

#### 2.3.3. Total Oxidative Stress (TOS) Assay

TOS levels of serum and aqueous were determined using commercial Rel Assay Diagnostic kits (Gaziantep, Turkey) with an autoanalyzer (Abbott, IL, USA). Oxidants present in the sample oxidize the ferrous ioneo-dianisidine complex to ferric ion. The reaction medium is rich in glycerol molecules that increase the oxidation reaction. A colored complex with xylenol orange in an acidic medium is produced by the ferric ion. The color intensity is associated with the amount of oxidant molecules present in the sample. The assay is calibrated with hydrogen peroxide and the outcomes are stated in terms of micromolar hydrogen peroxide equivalent per liter (*μ*mol H_2_O_2_ equiv./L).

#### 2.3.4. Total Antioxidant Status Assay

TAS was determined using commercial Rel Assay Diagnostic kits (Gaziantep, Turkey) with an autoanalyzer LX20-Pro from Beckman-Coulter (Woerden, The Netherlands). The method is based on the reduction of colored 2,2′-azino-bis radical to a colorless reduced form by the antioxidants. The absorbance is measured at 660 nm. The method is calibrated with the vitamin E analog (trolox equivalent) and the outcomes are expressed in mmol/L.

### 2.4. Statistical Analysis

Statistical analysis of the data was performed using SPSS (SPSS, Inc., Chicago, IL) for Windows 11.5 package program. Power analysis was performed by using G^*^Power v3.1.7 to detect the sample size. IMA, total thiol, TAC, and TOS levels in the serum and aqueous humor were compared statistically by using Mann-Whitney *U* test, Chi-square test, or Student's *t*-test in the two groups. Mean ± standard deviation (SD) or median and interquartile range values were used to describe quantitative data. Logistic regression analysis was used to determine the effects of IMA, total thiol, and TOS levels on DRP. The area beneath the receiver-operating characteristic (ROC) curves was also used to determine the discriminative power of IMA and total thiol levels in the diagnosis of DRP. Pearson product-moment and Spearman's rank correlation coefficients were calculated to determine the relationships between IMA, total thiol, TAS, and TOS levels. *P* < 0.05 was considered statistically significant.

## 3. Results

The patients' ages ranged from 55 to 79 years (mean: 65.54 ± 6.31) in DRP group and from 54 to 80 (mean: 65.72 ± 7.47) in the control group. Twenty-four patients were women and 20 patients were men. There is no statistically significant difference in terms of gender and age (*P* = 0.364 and 0.798, resp.).

Median serum IMA levels were 44.80 absorbance units in the DRP group compared with 40.15 absorbance units in the control group (*P* = 0.031). Median serum total thiol levels in the DRP group were statistically significantly less than those in the control group (3051.13 and 3910.12, resp., *P* = 0.004). Mean serum TAC levels were 1.46 ± 0.22 in the DRP group and 1.48 ± 0.21 in the control group (*P* = 0.828). Mean serum TOS levels were 2.93 ± 0.19 in the DRP group and 2.91 ± 0.26 in the control group (*P* = 0.039) (Table 1). There was no statistically significant correlation between IMA, total thiol, TAS, and TOS levels (*P* > 0.05).

Mean aqueous total thiol level was 263.15 ± 31.5 in the DRP group and 279.17 ± 40.65 in the control group (*P* = 0.152). Mean aqueous TAC level was 0.69 ± 0.09 in the DRP group and 0.65 ± 0.09 in the control group (*P* = 0.162). Mean aqueous TOS level was 290.73 ± 1.92 in the DRP group and 290.23 ± 1.26 in the control group (*P* = 0.313). The differences in mean aqueous total thiol, TAC, and TOS levels between the two groups were not statistically significant. IMA could not be detected in the aqueous (Table 2).

**Figure 1:**
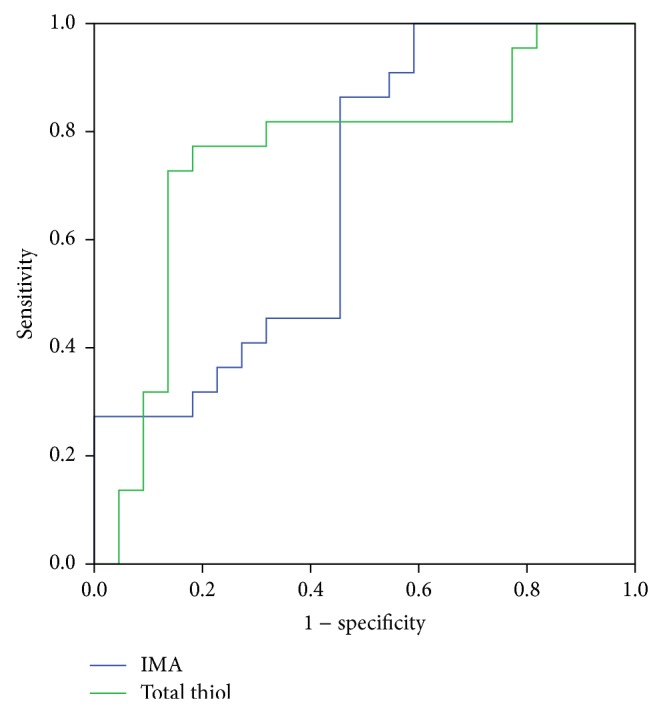
The ROC curves for IMA and total thiol in all diabetic patients.

The area beneath the receiver-operating characteristic (ROC) curves was also used to determine the discriminative power of IMA and total thiol levels in the anticipating of the development of DRP (Figure 1). The areas under the receiver-operating characteristic curves for the determination of DRP in patients with type 2 DM were 0.690 (%95 confidence interval: 0.533 to 0.821) for IMA and 0.756 (%95 CI: 0.603–0.873) for total thiol. The optimum diagnostic cutoff for IMA that increased specificity and sensitivity to the most in the estimation of retinopathy for all patients was 42 ABSU (50% and 86.6%, resp.; *P* = 0.031). This point was calculated as 3383.48 nmol/mL for serum total thiol level (77.3% and 81.8%, resp.; *P* = 0.004). Difference between areas under corresponding ROC curves was 0.066 and was found not to be statistically significant (*P* = 0.575). Some appropriate serum IMA and total thiol values obtained from the ROC curve, together with their specificity and sensitivity, are shown in Table 3.

In univariate logistic regression analysis, we found that the IMA, total thiol, and TOS levels in the serum of patients with type 2 DM were statistically significant risk factors for developing DRP (*P* = 0.003). When considering all risk factors together in multivariate logistic regression analyses, only IMA was statistically significant (Table 4).

## 4. Discussion

The results of our study reveal that the mean serum IMA and TOS levels in the DRP group were significantly higher than those in the control group, while the total thiol level was significantly lower. There was no statistically significant difference between the two groups regarding TAC levels in the serum of patients. Within the framework of the second stage of the study, we did not detect any IMA and there was no statistically significant difference regarding total thiol, TOS, and TAC in the aqueous humor of patients.

Ischemia plays a part in the pathogenesis of DRP and many other diseases [22]. Bar-Or et al. reported that the IMA concentration in the blood of patients, who had temporary ischemia because of percutaneous coronary angioplasty, started to elevate in a couple of minutes and when reperfusion was enabled by a subsequent angioplasty the IMA blood concentration, measured about 6 hours later, decreased to the levels of individuals with no ischemia [23]. The elevation in IMA concentration as an indicator of myocardial ischemia has been licensed by the Food and Drug Administration (FDA) in the evaluation of patients with coronary syndrome [24]. Furthermore, various studies have shown that serum IMA levels were significantly elevated in other diseases accompanied by ischemia such as systemic sclerosis [25], lower limb ischemia [26], pulmonary embolism [27], deep vein thrombosis [28], and strokes [29]. In light of the given information, IMA can be defined as a biomarker with a short half-life which is elevated in acute systemic conditions.

It was Piwowar et al. who first checked the IMA level of DM patients. The authors found higher IMA levels in diabetic patients than the healthy control group [9]. Ukinc et al. also pointed out to higher IMA levels in diabetic patients in comparison to healthy individuals in the control group. In addition, the authors reported that, in the existence of diabetic nephropathy, which is a vascular complication, IMA levels were higher than in diabetic patients with no nephropathy. The same study stressed the existence of chronic ischemia in diabetic patients and stated that high IMA levels might reflect an underlying subclinical vascular disease [10]. Turk et al. checked serum IMA levels in patients with DRP for the first time in the literature. The authors of the study concluded that the IMA levels of patients with DRP were higher than those of the patients in the control group. They also noted that IMA might reflect DRP as a result of ROC analyses related to IMA levels [12].

The results of our study indicated that the mean level of serum IMA in patients with type 2 DM was higher than in control subjects, in line with the results of other studies in the literature. Our study is the second one in which the serum IMA levels of patients with DRP were assessed following Turk et al. and it was also established that IMA levels were statistically higher in the DRP group. This result supports the idea that ischemia plays a role in the pathogenesis of DRP. Within the scope of our study, we also checked the IMA levels in aqueous humor in order to indicate the possible existence of aqueous barrier changes and chronic ischemia in the eye tissues of the DRP group but no IMA was found. The possible reasons why no IMA was found in the aqueous humor might be based on short IMA half-life, uninterrupted blood-aqueous barrier because all the DM patients had nonproliferative DRP, or the fact that the IMA concentration in aqueous might be below the detectable level. Our study is the first of its kind in which IMA levels in the aqueous humor of patients with DRP were studied.

Diabetes causes defects in metabolic pathways in relation to the affected antioxidant systems, increase in free radicals, and elevated glucose levels [12]. One of the antioxidant systems is the protein thiol groups [13]. Decreases and functional defects arise in the thiol groups as a result of the exposure of proteins to oxidative stress [13, 14]. Ceriello et al. compared the plasma total thiol levels of patients with type 2 DM followed up by diet or receiving oral antidiabetic treatment with those of the control group made up of healthy individuals and found that the total plasma thiol concentration was significantly low in the diabetic group [18]. Collier et al. also showed that the plasma thiol levels in patients with type 2 DM significantly decreased [30]. Yazıcı et al. stated that there was a decrease in plasma thiol levels, which is a sign of increase in oxidative stress in type II DM patients [31]. In our study, we checked the total thiol level, which is a significant part of the antioxidant system, in both aqueous humor and serum. Our study also accounts for the first study in the literature that investigated total thiol levels in serum and aqueous humor in DRP patients. The results of our study indicate that the serum total thiol level of the DRP group was significantly lower than that of the control group. We also found that the total thiol level of the DRP group in aqueous humor was lower than that of the control group but the difference between the two was not statistically significant. The result, which points out to the fact that total thiol (which is an antioxidant) decreased in DRP, supports the idea that the antioxidant system breaks down in DM.

Oxidative damage plays a role in the pathogenesis of many ocular degenerative diseases. Studies conducted on the subject proved the role of oxidative stress through investigating such signs as lipid peroxidation, antioxidant enzyme activity, and low molecular antioxidants [12]. In a study conducted in order to investigate the effects of oxidative stress on the progression of DRP, Uçgun et al. reported that serum TAC decreased while TOS increased in nonproliferative DRP and proliferative DRP groups in comparison to the control group [32]. Furthermore, Caner et al. found that the serum TAC value was significantly lower in DRP patients than the control group [33]. The same study also reported that the serum TOS value was significantly higher in the patient group than the control group [33]. In line with the literature, the results of our study indicated that serum TAC level was lower in the DRP group than the control group while the serum TOS level was higher. But the differences in TAC levels in the serum were not statistically significant. Caner et al. could not find a statistically significant difference between the groups regarding TAC and TOS levels in aqueous humor [33]. In our study, there was no statistically significant difference regarding total thiol, TOS, and TAC levels in the aqueous humor of patients.

As a result, our results support the idea that oxidative stress plays a role in the pathogenesis of DRP. Although the sample size is small, we demonstrate that serum IMA, total thiol, and TOS levels may be useful markers in monitoring the risk of DRP development. There is still a need, however, for comparative studies covering larger case series and diabetic patient groups with no retinopathy accompaniment together with proliferative DRP patients.

## Figures and Tables

**Table 1:** IMA, total thiol, TAC, and TOS levels in the serum.

	DRP group (*n* = 22)	Control group (*n* = 22)	*P* value
IMA (nm/l); median (*Q*1–*Q*3)	44.80 (43.60–77.40)	40.15 (20.55–56.83)	^ a^ **0.031**
Total thiol (*µ*mol/L); median (*Q*1–*Q*3)	3051.13 (2713.98–3370.43)	3910.12 (3421.95–4356.67)	^ a^ **0.004**
TAC mmol (trolox equiv./L); mean ± SD	1.46 ± 0.22	1.48 ± 0.21	^ b^ **0.828**
TOS (*µ*mol H_2_O_2_ equiv./L); mean ± SD	2.93 ± 0.19	2.91 ± 0.26	^ b^ **0.039**

^a^Mann-Whitney *U* test; ^b^Student's *t*-test; IMA: ischemia-modified albumin; TAC: total antioxidant capacity; TOS: total oxidative stress; *Q*: quartile.

**Table 2:** Mean IMA, total thiol, TAC, and TOS levels in the aqueous humor.

	DRP group (*n* = 22) Mean ± SD	Control group (*n* = 22) Mean ± SD	*P* value
Total thiol	263.15 ± 31.58	279.17 ± 40.65	**0.152** ^*^
TAC	0.69 ± 0.09	0.65 ± 0.09	**0.195** ^*^
TOS	290.73 ± 1.92	290.23 ± 1.26	**0.313** ^*^

^*^Student's *t*-test; IMA: ischemia-modified albumin; TAC: total antioxidant capacity; TOS: total oxidative stress.

**Table 3:** IMA and total thiol cutoff values and their specificity and sensitivity for prediction of DRP in all diabetic patients.

	Level	Sens.	Spec.	PPV	NPV	Acc.	Area	^ c^ *P*	DBA	^ d^ *P*
IMA	>42	86.36	50.00	63.33	78.57	68.18	0.690	0.031	0.066	0.575
Total thiol	≤3383	81.82	77.27	78.26	80.95	79.55	0.756	0.004		

^c^ *P*: significance levels of ROC curves; ^d^ *P*: significance level of difference between two ROC curves; Sens.: sensitivity; Spec.: specificity; PPV: positive predictive value; NPV: negative predictive value; Acc.: accuracy; DBA: difference between areas; IMA: ischemia-modified albumin.

**Table 4:** Results of multivariate logistic regression analysis.

	*P* value	Exp⁡(*B*)	95% CI for Exp⁡(*B*)	
			Lower	Upper
IMA	**0.045**	1.033	1.001	1.067
Total thiol	**0.117**	0.999	0.998	1.000
TOS	**0.151**	0.774	0.545	1.098

IMA: ischemia-modified albumin; TAC: total antioxidant capacity; TOS: total oxidative stress.
